# Quantitative Assessment of the Presence and Severity of Cirrhosis in Patients with Hepatitis B Using Right Liver Lobe Volume and Spleen Size Measured at Magnetic Resonance Imaging

**DOI:** 10.1371/journal.pone.0089973

**Published:** 2014-03-04

**Authors:** Xiao-li Chen, Tian-wu Chen, Xiao-ming Zhang, Zhen-lin Li, Nan-lin Zeng, Ting Li, Dan Wang, Jie Li, Zhi-jia Fang, Hang Li, Jia Chen, Jun Liu, Guo-hui Xu, Jing Ren, Jian-lin Wu, Chun-ping Li

**Affiliations:** 1 Sichuan Key Laboratory of Medical Imaging, and Department of Radiology, Affiliated Hospital of North Sichuan Medical College, Nanchong, Sichuan, China; 2 Department of Radiology, Sichuan Cancer Hospital and Institute & The Second People's Hospital of Sichuan Province, Chengdu, Sichuan, China; 3 Department of Radiology, West China Hospital of Sichuan University, Chengdu, Sichuan, China; 4 Department of Ultrasonography, Sichuan Academy of Medical Sciences & Sichuan Provincial People's Hospital, Chengdu, Sichuan, China; 5 Department of Radiology, Second Xiangya Hospital of Central South University, Changsha, Hunan, China; 6 Department of Radiology, Affiliated Zhongshan Hospital of Dalian University, Dalian, Liaoning, China; Wayne State University, United States of America

## Abstract

**Objective:**

To determine whether right liver lobe volume (RV) and spleen size measured utilizing magnetic resonance (MR) imaging could identify the presence and severity of cirrhosis in patients with hepatitis B.

**Methods:**

Two hundred and five consecutive patients with clinically confirmed diagnosis of cirrhosis due to hepatitis B and 40 healthy control individuals were enrolled in this study and underwent abdominal triphasic enhanced scans using MR imaging. Spleen maximal width (W), thickness (T) and length (L), together with RV and spleen volume (SV), were measured utilizing MR imaging. Spleen multidimensional index (SI) was obtained by multiplying previously acquired parameters W×T×L. Then statistical assessment was performed to evaluate the ability of these parameters, including RV, SV, RV/SV and SI, to identify the presence of cirrhosis and define Child-Pugh class of this disease.

**Results:**

SV and SI tended to increase (*r* = 0.557 and 0.622, respectively; all *P*<0.001), and RV and RV/SV tended to decrease (*r* = −0.749 and −0.699, respectively; all *P*<0.001) with increasing Child-Pugh class of cirrhosis. All the parameters, including RV, SV, RV/SV and SI, might be the indicators used to discriminate the patients with liver cirrhosis from the control group, and to distinguish these patients between Child-Pugh class A and B, between B and C, and between A and C (area under receiver operating characteristic curve [AUC] = 0.609–0.975, all *P*<0.05). Among these parameters, RV/SV was the best noninvasive factor for the discrimination of liver cirrhosis between Child-Pugh class A and B (AUC = 0.725), between A and C (AUC = 0.975), and between B and C (AUC = 0.876), while SI was the best variable to distinguish the cirrhosis patients from the control group (AUC = 0.960, *P*<0.05).

**Conclusion:**

RV/SV should be used to identify the severity of cirrhosis, while SI can be recommended to determine the presence of this disease.

## Introduction

Cirrhosis is an increasing cause of morbidity and mortality in more developed countries, being the 14th most common cause of death worldwide, and cirrhotic patients are at high risk for developing potentially fatal complications such as hepatic encephalopathy and liver failure during the progress of this disease [1]. In the assessment of the severity of liver cirrhosis, the modified Child-Pugh classification system is by far the most widely applied and reported system because it is easy to use as a bedside test. It divides the patients into low (class A), intermediate (class B), and poor (class C) risk within different levels between the least sick and the most advanced patients [2], [3]. The Child-Pugh classification is an independent prognostic factor for survival of cirrhosis patients, and stage C in the Child-Pugh classification is strongly associated with worse survival rates [2]–[4]. In addition, Child-Pugh classification scores must be taken into consideration for adequate evaluation of liver transplantation candidates [4].

Clinically, the most common signs and symptoms of patients with liver cirrhosis include liver dysfunction, splenomegaly and gastroesophageal varices. The change in the volume of the liver or spleen has been reported to correlate with the prognosis and severity of liver cirrhosis [5]–[8]. There is a large number of studies evaluating the correlation of splenomegaly with Child-Pugh scores, and clarifying the significance of splenomegaly in staging cirrhosis [5], [9], [10]. Additionally, the volume ratio of liver to spleen assessed with computed tomography could be an important indicator for predicting the development of symptoms and prognostic outcomes of the patients with liver cirrhosis [11]. Liu et al had reported that the volume ratio of liver to spleen was of a clinical value in the diagnosis of advanced liver fibrosis [12]. To our knowledge, there was no report about the utility of the combination of right liver lobe volume (RV) and spleen size to assess the presence of cirrhosis and define its Child-Pugh class.

Magnetic resonance (MR) imaging plays an increasingly important role in the assessment of liver diseases because it is the safe, effective and repeatable noninvasive modality [13], [14]. The purpose of this study was to investigate the feasibility of combination of RV and spleen size measured using MR imaging as well as the utility of these parameters alone to evaluate the presence and severity of liver cirrhosis, and to determine which parameter could best classify the severity of this disease.

## Materials and Methods

### Ethics statement

This study was approved by the institutional ethics review board of North Sichuan Medical College, and a written informed consent was obtained from each participant prior to the study.

### Participant selection

From February 2010 to March 2013, 298 consecutive patients were referred to Affiliated Hospital of North Sichuan Medical College according to the following inclusion criteria: (1) the diagnosis of cirrhosis in patients with hepatitis B was based on physical findings, laboratory investigations, image findings or histopathological findings whenever available, according to the American Association for the Study of Liver Diseases (AASLD) practice guidelines on chronic hepatitis B (2007) [15]; (2) the patients underwent abdominal triple-phase enhanced MR scans, and Child-Pugh score calculation using 5 parameters including albumin, ascites, bilirubin, prothrombin activity and encephalopathy [2], [3]; and (3) image data showed patients without portal vein emboli, hepatic artery-portal vein fistula, or hepatic carcinoma.

Exclusion criteria included the following: (1) patients had previous or active gastrointestinal bleeding (n = 35); (2) patients had a history of treatments for portal hypertension including splenectomy, partial spleen embolization, transjugular intrahepatic portosystemic shunt, balloon-occluded retrograde transvenous obliteration, β-blocker therapy, or endoscopic therapies (n = 43); (3) patients had concomitant hepatic schistosomiasis (n = 2); (4) patients had primary hematologic disorders (e.g., lymphoma and leukemia) (n = 6); or (5) patients had active alcohol abuse (less than six months of alcohol abstinence) (n = 7). Consequently, 205 patients were included in this study.

As for all the enrolled patients, the mean age was 56 years (range, 23–81 years), 127 patients (62%) were men, and 78 patients (38%) were woman. In this cohort, 72 patients (35.1%) had ascites and esophageal varices, 67 patients (32.7%) had ascites, 16 patients (7.8%) had esophageal varices, and 50 patients (24.4%) had neither ascites nor esophageal varices.

Additionally, a control group of 40 random consecutive healthy volunteers (24 men, 16 women; mean age 52 years; range, 21–76 years) who underwent upper abdominal triphasic enhancement MR scans at our institution served as the reference group used to obtain benchmarks. The inclusion criteria for the controls were no history of chronic liver disease, normal serum liver enzyme levels, and normal findings on abdominal MR scans. All volunteers had negative findings on hepatitis B and C surface antigen tests and laboratory examinations.

### MR technique

Participants in our study were examined on a 3.0 T whole body MR scanner (Signa; GE Medical Systems, Milwaukee, WI). When the respiratory signals were established, the patient was positioned supinely in an 8-channel phased array body coil. The routine MR sequences included SPGR T1-weighted imaging (T1WI), and fast recovery fast spin echo (FRFSE) T2-weighted imaging (T2WI). Subsequently, 10-mL gadodiamide (Magnevist; Bayer Healthcare, Germany) was intravenously injected via a pressure injector (Spectris MR Injection System; Medrad, Warrendale, PA) at a dose of 3 mL/s for a total of 0.2 mmol per kg of body weight followed by a 20-mL saline solution flush for axial contrast-enhanced three-dimensional liver acquisition with volume acceleration (3D-LAVA). The parameters for the axial 3D LAVA were as follows: TR = 3.9 ms, TE = 1.8 ms, field of view = 34×34 cm, slice thickness = 5.0 mm, and matrix of 256×224 mm. The scanning coverage was from the diaphragm to the inferior border of the spleen to cover the entire liver and spleen.

### Image analysis

**Figure 1:**
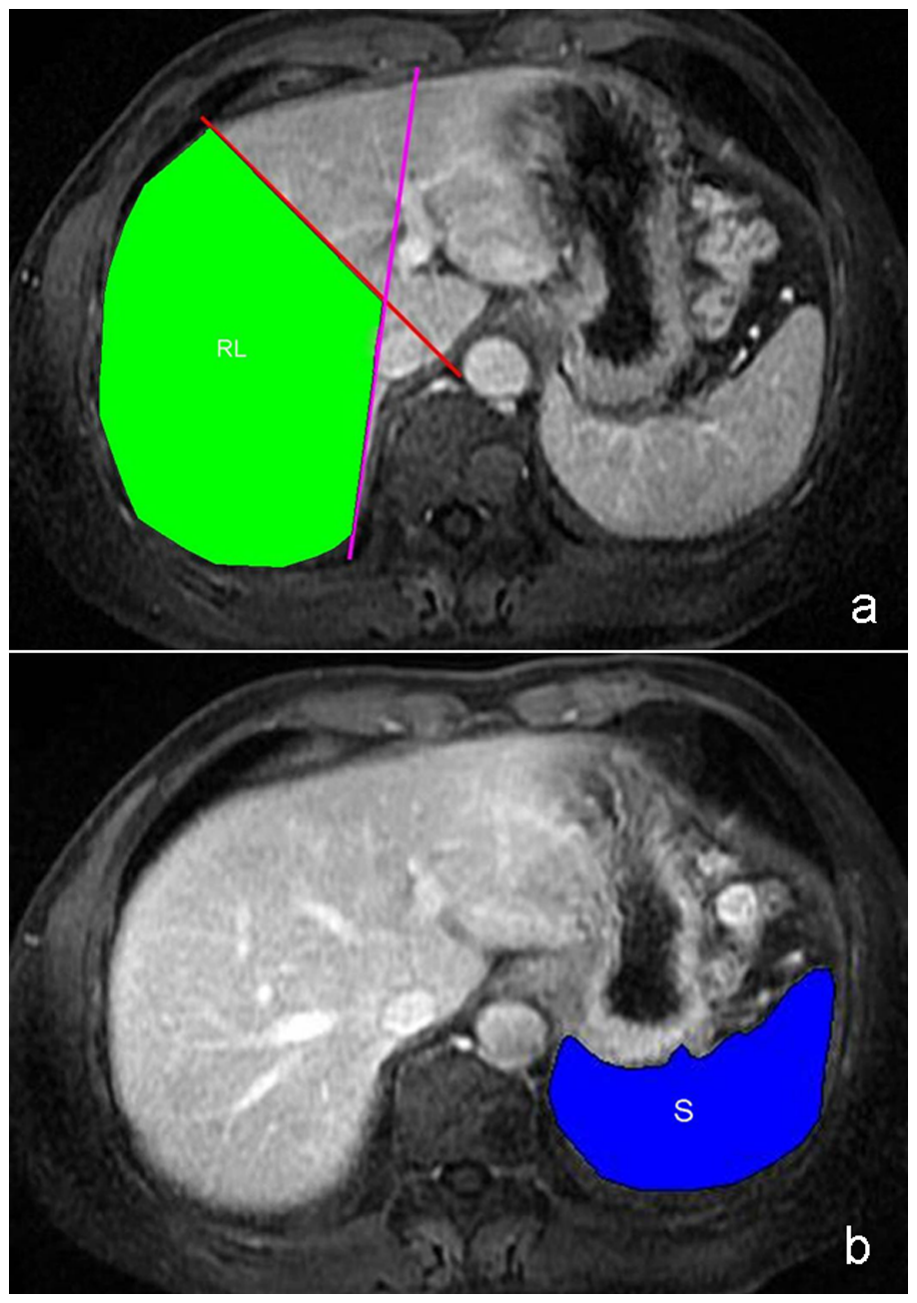
The outline of right liver lobe. Middle liver vein (red line) is used as a landmark to differentiate right liver lobe from left liver lobe, and the line linking the inferior vena cava to the right branch of the portal vein (pink line) is used as a landmark to differentiate right liver lobe from caudate lobe (a). Outline of right liver lobe (RL, a), and of spleen (S, b) are delineated on the axial enhanced magnetic resonance imaging.

The original MR data were directly interfaced and forwarded to the workstation (GE Advantage Workstation Version 4.4-09; Sun Microsystems, Palo Alto, CA, USA). The contrast-enhanced axial 3D-LAVA images were always presented first and in order that observers could identify the liver and spleen parenchyma. As described in the previous literature [6], [16], the portal venous phase images were used for the image analysis because the hepatic veins and inferior vena cava for tracing the boundaries of right liver lobe could be better depicted on the portal venous phase images than on arterial or delayed phase images. On the enhanced axial 3D-LAVA images, the RV and spleen volume (SV) were recorded independently by two radiologists (the corresponding author and the first author with 15 and 4 years of experiences in abdominal MR imaging, respectively) who were blinded to the clinical results. The RV and SV measurements were independently performed by using manual summation-of-area technique on cross-sectional images in each patient. According to the Goldsmith and Woodburne system, the liver was divided into four lobes: left lateral and medial lobes, right lobe and caudate lobe. To obtain the RV, the boundaries were manually traced on each axial section by using the computer's mouse until all contrast-enhanced images of right liver were covered, avoiding the areas of intrahepatic vasculature and gallbladder on each transverse image (Figure 1) [16], [17]. The software automatically calculated the number of pixels enclosed by the traced right liver lobe contour, and provided the cross-sectional area of the liver lobe on a slice-by-slice basis; and then the total outlined area was multiplied by the slice thickness to calculate RV [16], [17]. SV was obtained by a method similar to that of RV measurement. Subsequently, the ratio of RV to SV (RV/SV) was calculated. Three unidimensional measurements of spleen including spleen maximal width (W), thickness (T) and length (L) as described previously were recorded independently by the above-mentioned radiologists. Spleen W was recorded as maximum width on axial sections of the spleen, spleen T was defined as the maximum thickness of spleen on axial images, and spleen L was obtained by multiplying the number of sections where the spleen was visualized by the thickness of an axial section [18]. As previously described, the W was multiplied by the T, and this product was then multiplied by the L (W×T×L) in order to obtain spleen index (SI).

### Statistical analyses

All statistical analysis was carried out with SPSS (version 17.0, SPSS, Chicago IL, USA). A *P* value of less than 0.05 was considered to represent significant difference.

The interobserver agreement in the RV and spleen size parameters (W, T, L and SV) of the two independent observers' measurements was assessed using interclass correlation coefficient (ICC) mode in addition to 95% confidence interval (95%CI).

The univariate analysis was performed to evaluate the association of the MR parameters (RV, SV, SI and RV/SV) and possible clinical variables with the presence of cirrhosis by using the Chi-square tests. Spearman's rank correlation analysis was used to assess the correlations between the potential identifying parameters and the Child-Pugh class of liver cirrhosis. To evaluate the statistical differences in each of the MR parameters among the Child-Pugh classes of liver cirrhosis, the multiple pairwise comparisons were conducted with the Mann-Whitney U test with Bonferroni correction. The cutoff values of RV, SV, SI and RV/SV were then determined by receiver operating characteristic (ROC) analysis with the area under the ROC curve (AUC) for the identification of the Child-Pugh class of liver cirrhosis.

## Results

### Interobserver measurements agreement

In all the enrolled patients and healthy volunteers, there were good agreements between the measurements of the two observers for W (ICC, 0.96; 95%CI, 0.92–0.99), T (ICC, 0.95; 95%CI, 0.90–0.99), L (ICC, 0.92; 95%CI, 0.86–0.98), SV (ICC, 0.93; 95% CI, 0.90–0.99), and RV (ICC, 0.88; 95% CI, 0.81–0.95), and the first observer measurements were used as the final results.

### Associations of the MR and possible clinical variables with the presence and Child-Pugh class of cirrhosis

The gender, age, body weight, body mass index, RV, SV, SI and RV/SV of all the participants are shown in Table 1. The univariate analysis showed that cirrhosis patients were more likely to have lower RV and RV/SV, and larger SV and SI than the control group volunteers (all *P*<0.001). However, there was no significant difference in gender (*P* = 0.816), age (*P* = 0.056), body weight (*P* = 0.095), or body mass index (*P* = 0.076) between patients and volunteers.

**Table 1:** The main clinical characteristics of the healthy volunteers and patients with cirrhosis in different modified Child-Pugh class.

	Volunteers (n = 40)	Patients with Cirrhosis		
		Class A (n = 47)	Class B (n = 95)	Class C (n = 63)
Gender (M/F)	24/16	24/23	60/35	43/20
Age	51.50±2.31	58.13±1.83	55.99±1.18	54.38±1.63
Body weight (kg)	61.98±1.98	59.3±2.07	58.23±1.06	55.41±1.35
BMI (kg/m^2^)	21.44±0.63	20.52±0.31	20.15±0.27	19.17±0.30
RV (mm^3^)	945.3±14.27	809.69±27.29	696.81±13.93^a^	515.10±14.60^a^ ^b^
SV (mm^3^)	143.11±9.58	299.87±27.78	429.82±60.74^a^	854.60±208.57^a^ ^b^
SI (mm^3^)	287.61±17.72	649.09±62.52	1022.72±149.35^a^	2432.04±504.62^a^ ^b^
RV/SV	7.98±0.59	3.57±0.27	2.53±0.18^a^	0.92±0.07^a^ ^b^

Notes: BMI = body mass index, SV = spleen volume, SI = spleen maximal width×thickness×length, and RV = right liver volume.

a different from class A, and

b different from class B; and all the comparisons denotes significance after Bonferroni correction (*P*<0.05).

Spearman's rank correlation analysis illustrated that there was a trend toward increasing in SV and SI with the increasing Child-Pugh class (*r* = 0.557 and 0.622, respectively; all *P*<0.001). Furthermore, the multiple pairwise comparisons demonstrated that SV and SI were significantly higher in Child-Pugh class B than in class A (*P* = 0.035 and 0.004, respectively), and in class C than in class B (all *P*<0.001) or A (all *P*<0.001). With the increasing class of Child-Pugh, there was a trend toward decreasing in RV and RV/SV (*r* = −0.749 and −0.699, respectively; all *P*<0.001). There were significant differences in RV and RV/SV between Child-Pugh class A and B, between A and C, or between B and C (all *P*<0.001).

### ROC analysis of the MR variables for identifying Child-Pugh class of cirrhosis

**Figure 2:**
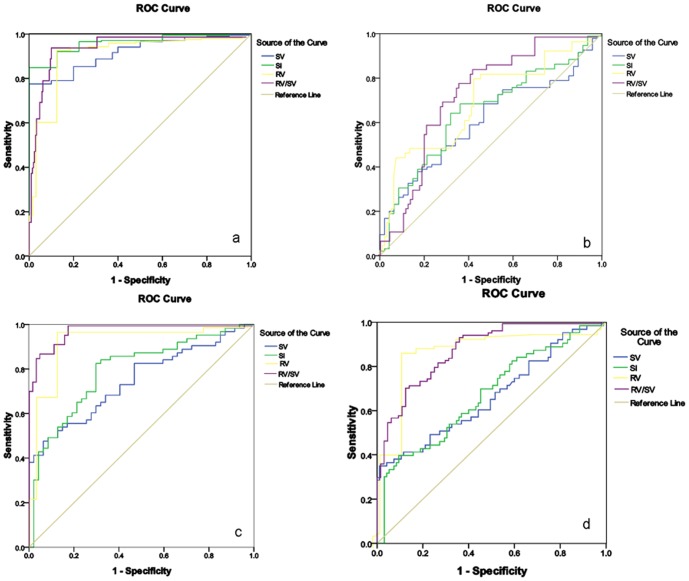
Receiver operating characteristic curves of the magnetic resonance variables for identifying Child-Pugh class of cirrhosis. The figures show that the parameters including right liver lobe volume (RV), spleen volume (SV), spleen multidimensional index (SI) and RV/SV are indicators for the discrimination between cirrhosis patients and volunteers (a); and for classifying cirrhosis between Child-Pugh class A and B (b), between A and C (c), and between B and C (d).

The cutoff values, sensitivity and specificity of RV, SV, SI and RV/SV for differentiating between cirrhosis patients and volunteers, or among Child-Pugh classes of cirrhosis are shown in Table 2. All the parameters might be the indicators for the discrimination between cirrhosis patients and control group volunteers; and for identifying cirrhosis between Child-Pugh class A and B, between B and C, and between A and C (Figure 2).

**Table 2:** Receiver operating curve analysis of the magnetic resonance variables for determining the presence and Child-Pugh class of liver cirrhosis.

Parameters	Cut-off value	Differentiations	AUC	Sensitivity	Specificity
RV (mm^3^)	878.25	Normal liver vs. cirrhosis	0.916	95%	92.7%
	720.50	Class A vs. B	0.696	61.7%	61.1%
	594.20	Class A vs. C	0.930	97.9%	87.2%
	567.50	Class B vs. C	0.869	90.5%	81%
SV (mm^3^)	248.10	Normal liver vs. cirrhosis	0.916	77.6%	100%
	399.90	Class A vs. B	0.609	52.6%	66%
	405.10	Class A vs. C	0.747	68.3%	66%
	534.35	Class B vs. C	0.665	54%	67.4%
SI (mm^3^)	545.03	Normal liver vs. cirrhosis	0.960	84.9%	100%
	774.50	A vs. B	0.649	68.4%	63.8%
	904.10	A vs. C	0.793	82.5%	70.2%
	1202.85	B vs. C	0.671	60.3%	60%
RV/SV	4.09	Normal liver vs. cirrhosis	0.951	95%	89.8%
	2.73	Class A vs. B	0.725	70.2%	70.5%
	1.92	Class A vs. C	0.975	87.2%	95.2%
	1.25	Class B vs. C	0.876	82.1%	73%

Notes: SV = spleen volume, RV = right liver volume, and SI = spleen width×thickness×length. AUC = area under the receiver operating curve.

As shown in Table 2, RV/SV had a larger AUC than either RV or SV alone in the discrimination between liver cirrhosis patients and volunteers, and the classification among Child-Pugh classes of liver cirrhosis. Among all the MR parameters, SI obtained the largest AUC for the discrimination between liver cirrhosis patients and volunteers, and RV/SV had the largest AUC for the discrimination of liver cirrhosis between Child-Pugh class A and B, between A and C, or between B and C (Figure 2).

## Discussion

In recent years, the availability of noninvasive tools has increased the proportion of patients with chronic liver cirrhosis diagnosed at the compensated or decompensated stage [4]–[11]. As one of the noninvasive tools, MR imaging can provide accurate three-dimensional reconstruction images, and then fine anatomic images of each liver lobe or spleen could be obtained even in patients with significant ascites, which could achieve the purpose of accurate measurement of the volumes of liver lobe or spleen [19]. In view of the decrease of RV and increase of spleen size with the progress of liver cirrhosis, we performed this current study to compare the diagnostic efficiency of simple available parameters based on RV and the spleen size with that of the combined parameter RV/SV obtained at MR imaging in identifying the presence and Child-Pugh class of liver cirrhosis.

As shown in our study, there was a trend toward decreasing in RV with the increasing Child-Pugh class of cirrhosis. In the typical cirrhosis, the liver volume, especially the RV, is significantly narrowed, which is closely related to the blood perfusion of portal vein. The right portal vein is directly into the right liver lobe parenchyma, while the liver fibrosis and cirrhosis nodules increase the intrahepatic portal vein pressure and irregular stenosis, and reduce liver vascular bed area and its blood flow, resulting in the obvious atrophy of right liver lobe [20], [21]. As cirrhosis progresses, this hepatic morphologic change including shrinkage of the right lobe in particular often occurs [22], [23].

In this study, we found that there was a trend toward increasing in SV and SI with the increasing Child-Pugh class of liver cirrhosis. Our results were consistent with the published reports, which showed SV increasing with the progress of liver fibrosis and cirrhosis [19], [24]. This finding could be explained by the progressive increase in intrahepatic portal vein pressure gradient with increasing severity of liver cirrhosis as shown in the histological-hemodynamic studies [25]. The resultant increase in the portal vein pressure may increase the barriers to spleen blood stream, and then spleen enlarges with the increasing portal vein diameter, splenic blood flow and portal blood flow [10], [26], [27]. Therefore, the portal hemodynamics may be important in splenomegaly resulted from cirrhosis [28].

Additionally, we reported that there was a trend toward decreasing in RV/SV with the increasing Child-Pugh class of liver cirrhosis. This finding can be explained by the possible pathologic mechanism that patients with liver cirrhosis are often presented with the atrophy of right liver lobe, and splenomegaly. We have confirmed that, in cirrhosis, the liver is not the only one organ to be mainly characterized by diminution, and that the spleen enlarges with the increasing spleen blood flow [10], [16]. For the first time, we combined SV and RV in this study to quantitatively assess the presence and severity of liver cirrhosis. In addition, we did not combine SI and RV to evaluate the progress of liver cirrhosis because SI was not as accurate as SV to depict the spleen size.

Because of significant trends toward increasing in SV and SI and decreasing in RV and RV/SV with the progress of liver cirrhosis, we performed the diagnostic analysis by using all the parameters to differentiate between cirrhosis patients and volunteers, and to identify the Child-Pugh class of liver cirrhosis. We confirmed that SV, SI, RV and RV/SV could be used to identify the presence and severity of cirrhosis. We also found that the performance of the combined parameters (RV/SV) was clearly superior to either RV or SV for identifying the occurrence and Child-Pugh class of liver cirrhosis because a better AUC could be obtained from the combined parameters than from either single parameter. Among these parameters, SI was the best single variable for the discrimination of liver cirrhosis patients from volunteers, which may be due to the fact that SI enlarges the efficiency of this discrimination in comparison with SV. Most importantly, we confirmed that RV/SV was the best parameter for the discrimination of liver cirrhosis between Child-Pugh class A and B, between A and C, and between B and C. Therefore, RV/SV can be recommended as a suitable indicator for identifying the severity of liver cirrhosis after the presence of this disease is determined with SI.

There was a limitation in our study. Liver cirrhosis was not confirmed by aspiration biopsy in some enrolled patients because these patients had abnormal coagulation. To overcome this limitation, each patient received the clinical assessment of Child-Pugh class score because this scoring system was popular for evaluating the severity of cirrhosis. In addition, the diagnosis of liver cirrhosis in these patients with hepatitis B was made according to the American Association for the Study of Liver Diseases (AASLD) practice guidelines on chronic hepatitis B (2007) [15].

In conclusion, the combined parameters of SV and RV are superior to either single parameter to identify the occurrence and severity of cirrhosis. RV/SV can be an appropriate indicator for identifying the severity of liver cirrhosis while SI can be a sensitive indicator for determining the occurrence of this disease. We hope that the recommended noninvasive parameters can improve the sensitivity and accuracy of the detection and classification of liver cirrhosis.
